# Supplementary material for: Differential PaxillinB dynamics at Dictyostelium cell-substrate adhesions
Source: bioRxiv. 2025 Aug 6:2025.08.04.668536. Preprint. [Version 1] doi: 10.1101/2025.08.04.668536 (PMC12340833; doi:10.1101/2025.08.04.668536)
Supplement: Supplement 1 [file media-1.pdf]

SUPPLEMENTARY INFORMATION

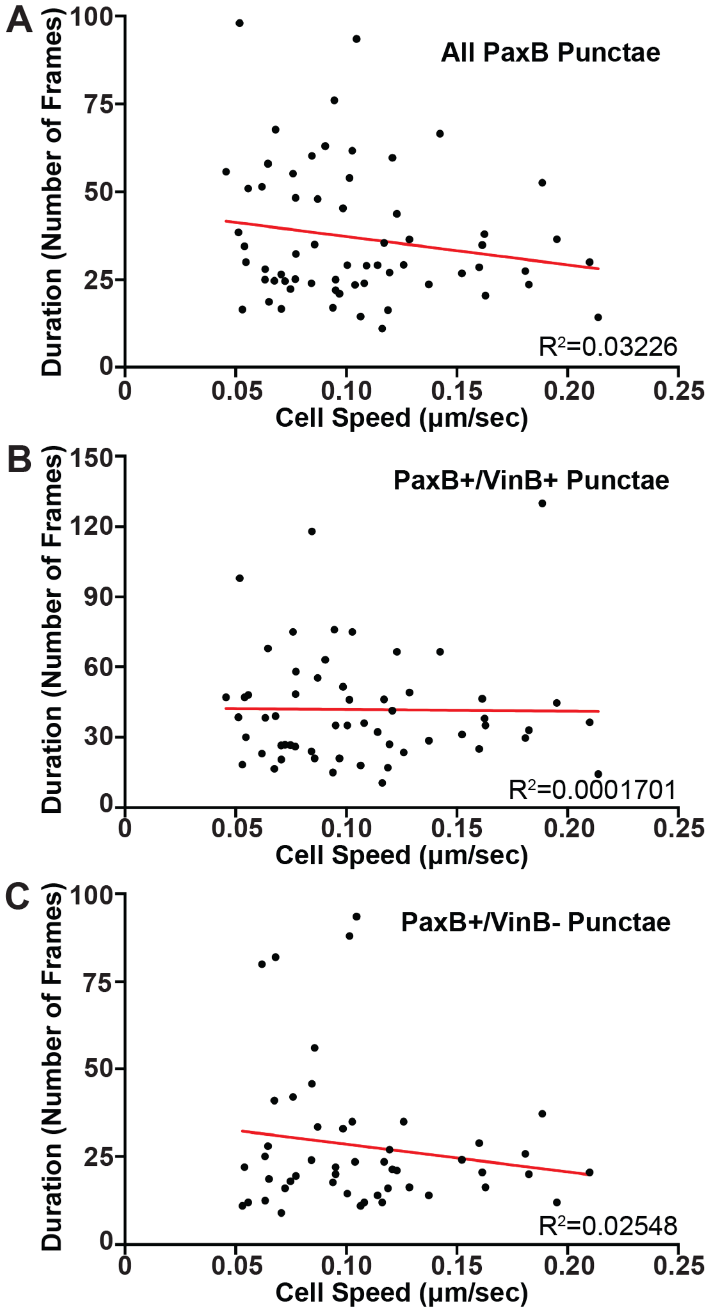

Supplemental Figure 1: *Dictyostelium* cell-substrate adhesion duration does not correlate with cell migration speed

Scatterplot graphs comparing cell migration speed to the duration of **A)** PaxillinB-positive punctae, **B)** PaxillinB+/VinculinB+ punctae or **C)** PaxillinB+/VinculinB- punctae across n = 65 (A), 57 (B), and 48 (C) cells, respectively. Correlation analyses of cell migration speed and punctae duration suggest there is no correlation between punctae duration and cell migration speed regardless of adhesion composition.
